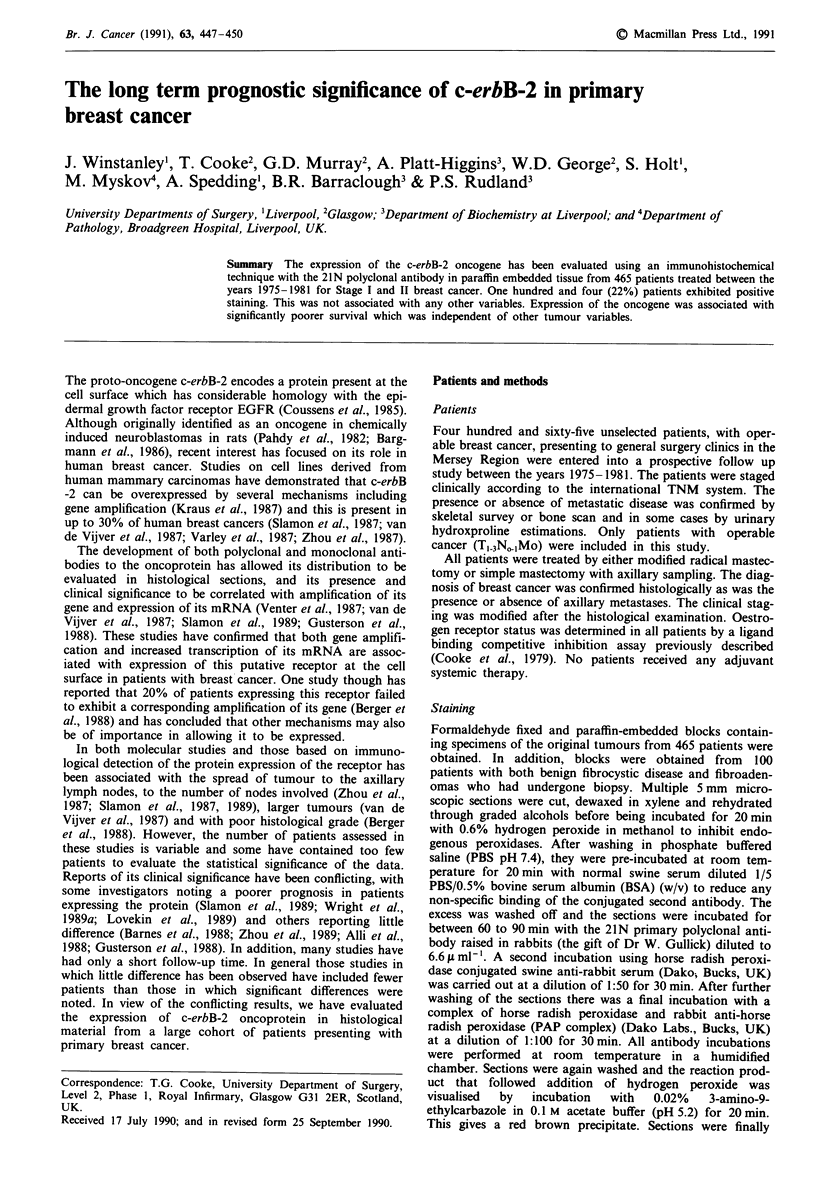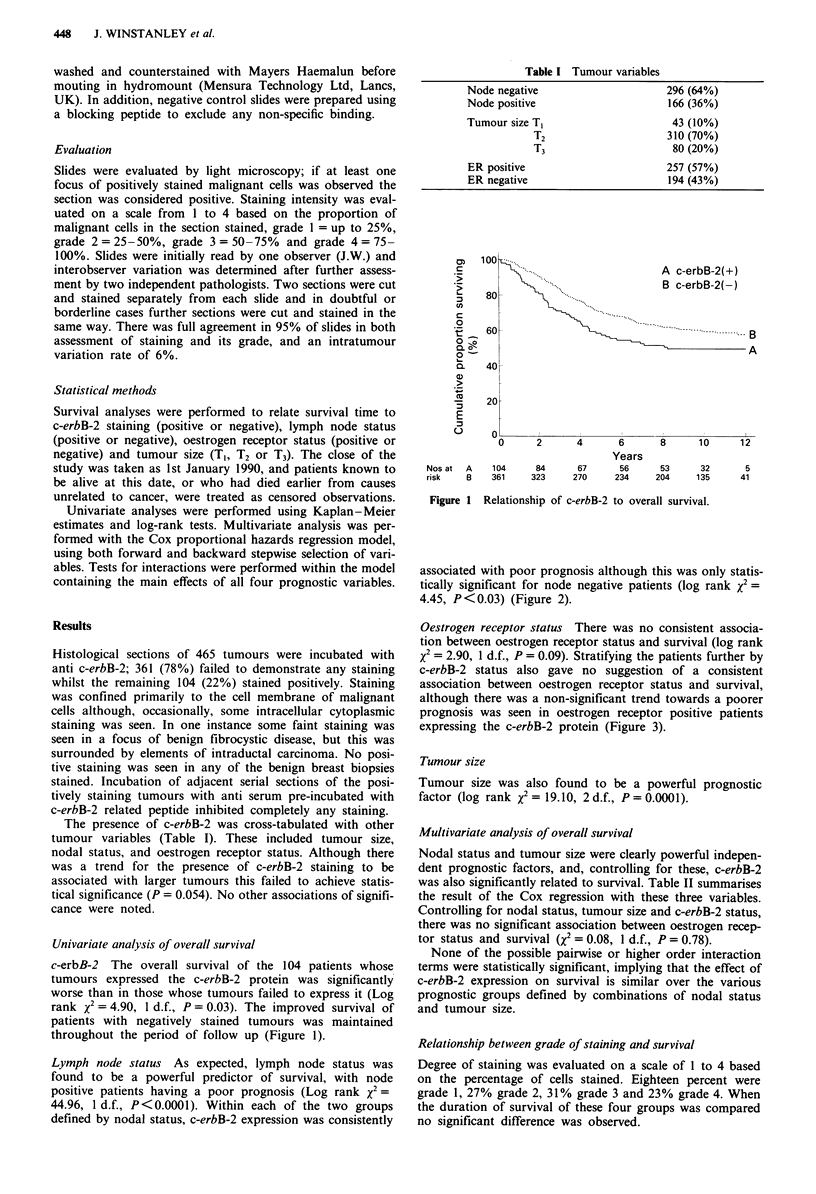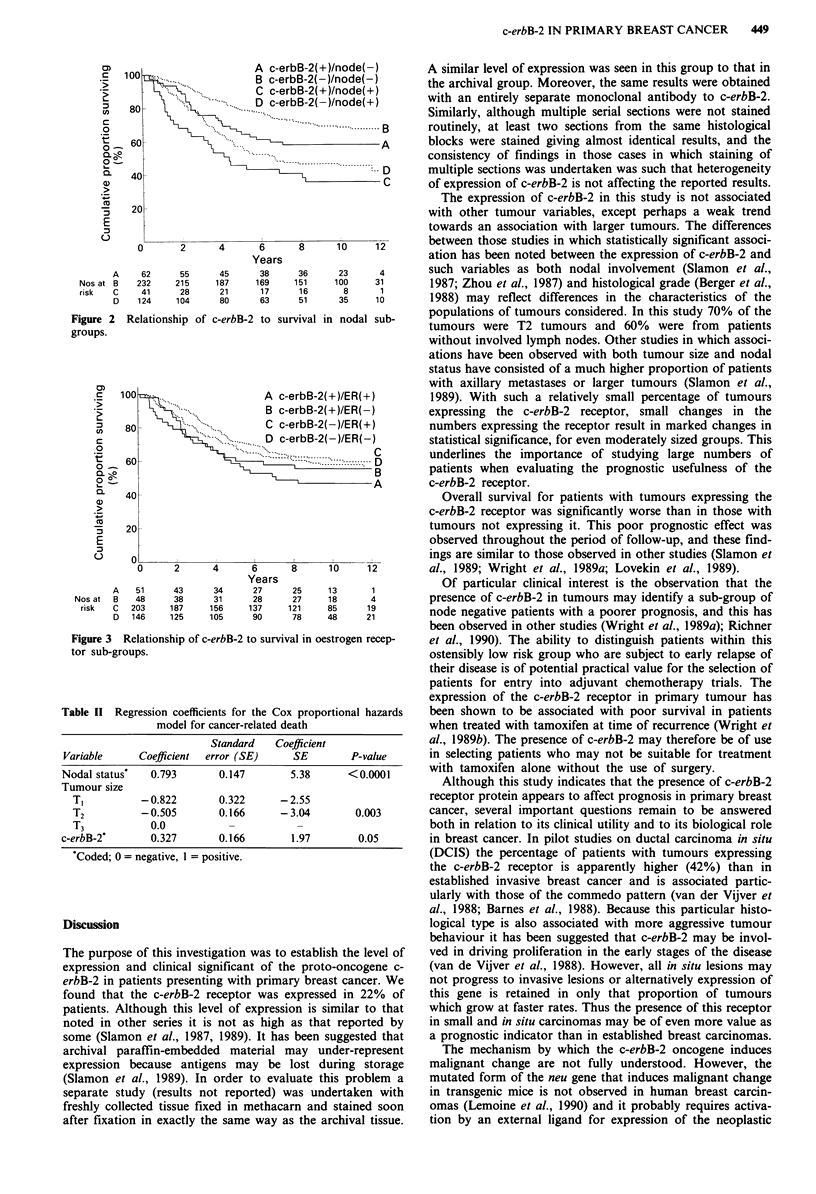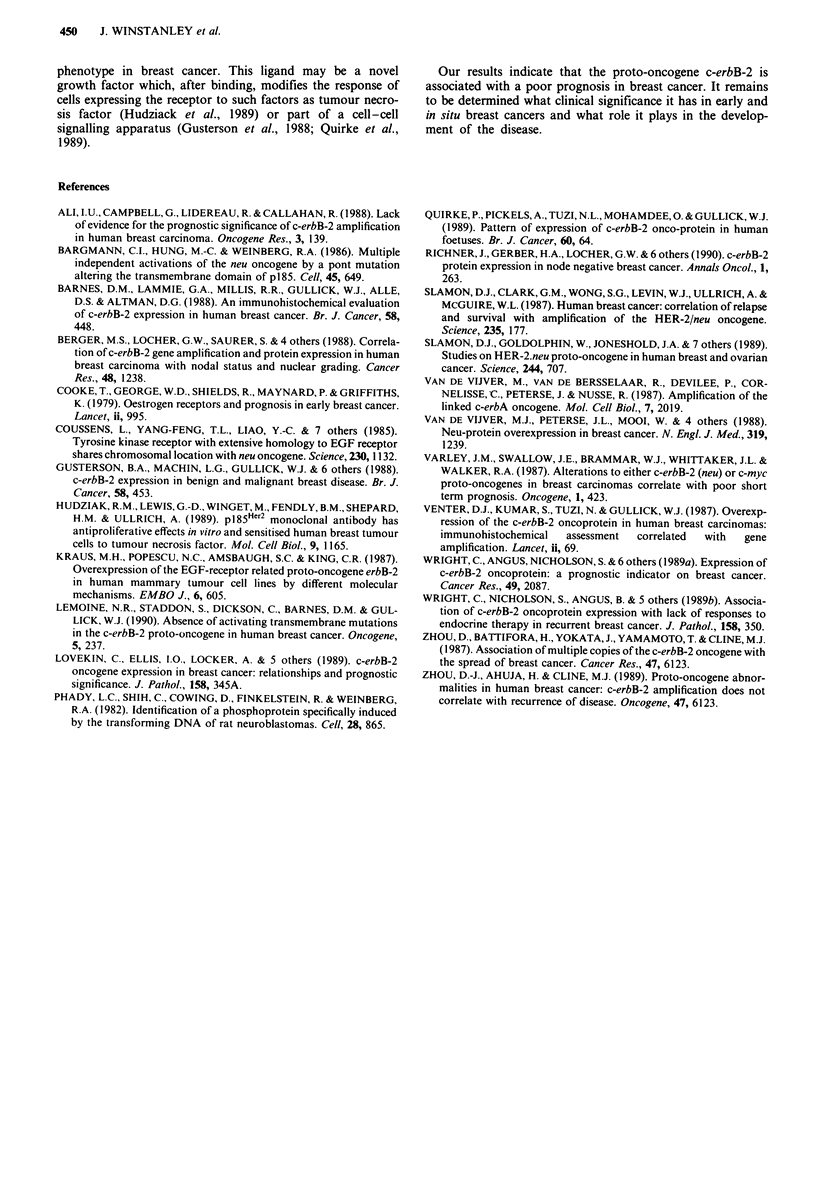# The long term prognostic significance of c-erbB-2 in primary breast cancer.

**DOI:** 10.1038/bjc.1991.103

**Published:** 1991-03

**Authors:** J. Winstanley, T. Cooke, G. D. Murray, A. Platt-Higgins, W. D. George, S. Holt, M. Myskov, A. Spedding, B. R. Barraclough, P. S. Rudland

**Affiliations:** University Department of Surgery, Liverpool, UK.

## Abstract

The expression of the c-erbB-2 oncogene has been evaluated using an immunohistochemical technique with the 21N polyclonal antibody in paraffin embedded tissue from 465 patients treated between the years 1975-1981 for Stage I and II breast cancer. One hundred and four (22%) patients exhibited positive staining. This was not associated with any other variables. Expression of the oncogene was associated with significantly poorer survival which was independent of other tumour variables.


Br. J. Cancer (1991), 63, 447-450 © Macmillan Press Ltd., 1991

### The long term prognostic significance of c-*erb*B-2 in primary breast cancer

J. Winstanley[1], T. Cooke[2], G.D. Murray[2], A. Platt-Higgins[3], W.D. George[2], S. Holt[1], M. Myskov[4], A. Spedding[1], B.R. Barraclough[3] & P.S. Rudland[3]

*University Departments of Surgery, [1]Liverpool, [2]Glasgow; [3]Department of Biochemistry at Liverpool; and [4]Department of Pathology, Broadgreen Hospital, Liverpool, UK.*

**Summary** The expression of the c-*erb*B-2 oncogene has been evaluated using an immunohistochemical technique with the 21N polyclonal antibody in paraffin embedded tissue from 465 patients treated between the years 1975-1981 for Stage I and II breast cancer. One hundred and four (22%) patients exhibited positive staining. This was not associated with any other variables. Expression of the oncogene was associated with significantly poorer survival which was independent of other tumour variables.

The proto-oncogene c-*erb*B-2 encodes a protein present at the cell surface which has considerable homology with the epidermal growth factor receptor EGFR (Coussens *et al.*, 1985). Although originally identified as an oncogene in chemically induced neuroblastomas in rats (Pahdy *et al.*, 1982; Bargmann *et al.*, 1986), recent interest has focused on its role in human breast cancer. Studies on cell lines derived from human mammary carcinomas have demonstrated that c-*erb*B -2 can be overexpressed by several mechanisms including gene amplification (Kraus *et al.*, 1987) and this is present in up to 30% of human breast cancers (Slamon *et al.*, 1987; van de Vijver *et al.*, 1987; Varley *et al.*, 1987; Zhou *et al.*, 1987).

The development of both polyclonal and monoclonal antibodies to the oncoprotein has allowed its distribution to be evaluated in histological sections, and its presence and clinical significance to be correlated with amplification of its gene and expression of its mRNA (Venter *et al.*, 1987; van de Vijver *et al.*, 1987; Slamon *et al.*, 1989; Gusterson *et al.*, 1988). These studies have confirmed that both gene amplification and increased transcription of its mRNA are associated with expression of this putative receptor at the cell surface in patients with breast cancer. One study though has reported that 20% of patients expressing this receptor failed to exhibit a corresponding amplification of its gene (Berger *et al.*, 1988) and has concluded that other mechanisms may also be of importance in allowing it to be expressed.

In both molecular studies and those based on immunological detection of the protein expression of the receptor has been associated with the spread of tumour to the axillary lymph nodes, to the number of nodes involved (Zhou *et al.*, 1987; Slamon *et al.*, 1987, 1989), larger tumours (van de Vijver *et al.*, 1987) and with poor histological grade (Berger *et al.*, 1988). However, the number of patients assessed in these studies is variable and some have contained too few patients to evaluate the statistical significance of the data. Reports of its clinical significance have been conflicting, with some investigators noting a poorer prognosis in patients expressing the protein (Slamon *et al.*, 1989; Wright *et al.*, 1989*a*; Lovekin *et al.*, 1989) and others reporting little difference (Barnes *et al.*, 1988; Zhou *et al.*, 1989; Alli *et al.*, 1988; Gusterson *et al.*, 1988). In addition, many studies have had only a short follow-up time. In general those studies in which little difference has been observed have included fewer patients than those in which significant differences were noted. In view of the conflicting results, we have evaluated the expression of c-*erb*B-2 oncoprotein in histological material from a large cohort of patients presenting with primary breast cancer.

Correspondence: T.G. Cooke, University Department of Surgery, Level 2, Phase 1, Royal Infirmary, Glasgow G31 2ER, Scotland, UK.

Received 17 July 1990; and in revised form 25 September 1990.

### Patients and methods

### *Patients*

Four hundred and sixty-five unselected patients, with operable breast cancer, presenting to general surgery clinics in the Mersey Region were entered into a prospective follow up study between the years 1975-1981. The patients were staged clinically according to the international TNM system. The presence or absence of metastatic disease was confirmed by skeletal survey or bone scan and in some cases by urinary hydroxproline estimations. Only patients with operable cancer ($T_{1\text{-}3}N_{0\text{-}1}Mo$) were included in this study.

All patients were treated by either modified radical mastectomy or simple mastectomy with axillary sampling. The diagnosis of breast cancer was confirmed histologically as was the presence or absence of axillary metastases. The clinical staging was modified after the histological examination. Oestrogen receptor status was determined in all patients by a ligand binding competitive inhibition assay previously described (Cooke *et al.*, 1979). No patients received any adjuvant systemic therapy.

### *Staining*

Formaldehyde fixed and paraffin-embedded blocks containing specimens of the original tumours from 465 patients were obtained. In addition, blocks were obtained from 100 patients with both benign fibrocystic disease and fibroadenomas who had undergone biopsy. Multiple 5 mm microscopic sections were cut, dewaxed in xylene and rehydrated through graded alcohols before being incubated for 20 min with 0.6% hydrogen peroxide in methanol to inhibit endogenous peroxidases. After washing in phosphate buffered saline (PBS pH 7.4), they were pre-incubated at room temperature for 20 min with normal swine serum diluted 1/5 PBS/0.5% bovine serum albumin (BSA) (w/v) to reduce any non-specific binding of the conjugated second antibody. The excess was washed off and the sections were incubated for between 60 to 90 min with the 21N primary polyclonal antibody raised in rabbits (the gift of Dr W. Gullick) diluted to 6.6 $\mu$ ml$^{-1}$. A second incubation using horse radish peroxidase conjugated swine anti-rabbit serum (Dako, Bucks, UK) was carried out at a dilution of 1:50 for 30 min. After further washing of the sections there was a final incubation with a complex of horse radish peroxidase and rabbit anti-horse radish peroxidase (PAP complex) (Dako Labs., Bucks, UK) at a dilution of 1:100 for 30 min. All antibody incubations were performed at room temperature in a humidified chamber. Sections were again washed and the reaction product that followed addition of hydrogen peroxide was visualised by incubation with 0.02% 3-amino-9-ethylcarbazole in 0.1 M acetate buffer (pH 5.2) for 20 min. This gives a red brown precipitate. Sections were finally washed and counterstained with Mayers Haemalun before mouting in hydromount (Mensura Technology Ltd, Lancs, UK). In addition, negative control slides were prepared using a blocking peptide to exclude any non-specific binding.

*Evaluation*

Slides were evaluated by light microscopy; if at least one focus of positively stained malignant cells was observed the section was considered positive. Staining intensity was evaluated on a scale from 1 to 4 based on the proportion of malignant cells in the section stained, grade 1 = up to 25%, grade 2 = 25–50%, grade 3 = 50–75% and grade 4 = 75–100%. Slides were initially read by one observer (J.W.) and interobserver variation was determined after further assessment by two independent pathologists. Two sections were cut and stained separately from each slide and in doubtful or borderline cases further sections were cut and stained in the same way. There was full agreement in 95% of slides in both assessment of staining and its grade, and an intratumour variation rate of 6%.

*Statistical methods*

Survival analyses were performed to relate survival time to c-*erb*B-2 staining (positive or negative), lymph node status (positive or negative), oestrogen receptor status (positive or negative) and tumour size ($T_1$, $T_2$ or $T_3$). The close of the study was taken as 1st January 1990, and patients known to be alive at this date, or who had died earlier from causes unrelated to cancer, were treated as censored observations.

Univariate analyses were performed using Kaplan–Meier estimates and log-rank tests. Multivariate analysis was performed with the Cox proportional hazards regression model, using both forward and backward stepwise selection of variables. Tests for interactions were performed within the model containing the main effects of all four prognostic variables.

### Results

Histological sections of 465 tumours were incubated with anti c-*erb*B-2; 361 (78%) failed to demonstrate any staining whilst the remaining 104 (22%) stained positively. Staining was confined primarily to the cell membrane of malignant cells although, occasionally, some intracellular cytoplasmic staining was seen. In one instance some faint staining was seen in a focus of benign fibrocystic disease, but this was surrounded by elements of intraductal carcinoma. No positive staining was seen in any of the benign breast biopsies stained. Incubation of adjacent serial sections of the positively staining tumours with anti serum pre-incubated with c-*erb*B-2 related peptide inhibited completely any staining.

The presence of c-*erb*B-2 was cross-tabulated with other tumour variables (Table I). These included tumour size, nodal status, and oestrogen receptor status. Although there was a trend for the presence of c-*erb*B-2 staining to be associated with larger tumours this failed to achieve statistical significance ($P = 0.054$). No other associations of significance were noted.

*Univariate analysis of overall survival*

*c-*erb*B-2* The overall survival of the 104 patients whose tumours expressed the c-*erb*B-2 protein was significantly worse than in those whose tumours failed to express it (Log rank $\chi^2 = 4.90$, 1 d.f., $P = 0.03$). The improved survival of patients with negatively stained tumours was maintained throughout the period of follow up (Figure 1).

*Lymph node status* As expected, lymph node status was found to be a powerful predictor of survival, with node positive patients having a poor prognosis (Log rank $\chi^2 = 44.96$, 1 d.f., $P < 0.0001$). Within each of the two groups defined by nodal status, c-*erb*B-2 expression was consistently

**Table I** Tumour variables

| | |
|---|---|
| Node negative | 296 (64%) |
| Node positive | 166 (36%) |
| Tumour size $T_1$ | 43 (10%) |
| $T_2$ | 310 (70%) |
| $T_3$ | 80 (20%) |
| ER positive | 257 (57%) |
| ER negative | 194 (43%) |

**Figure 1** Relationship of c-*erb*B-2 to overall survival.

associated with poor prognosis although this was only statistically significant for node negative patients (log rank $\chi^2 = 4.45$, $P < 0.03$) (Figure 2).

*Oestrogen receptor status* There was no consistent association between oestrogen receptor status and survival (log rank $\chi^2 = 2.90$, 1 d.f., $P = 0.09$). Stratifying the patients further by c-*erb*B-2 status also gave no suggestion of a consistent association between oestrogen receptor status and survival, although there was a non-significant trend towards a poorer prognosis was seen in oestrogen receptor positive patients expressing the c-*erb*B-2 protein (Figure 3).

*Tumour size*

Tumour size was also found to be a powerful prognostic factor (log rank $\chi^2 = 19.10$, 2 d.f., $P = 0.0001$).

*Multivariate analysis of overall survival*

Nodal status and tumour size were clearly powerful independent prognostic factors, and, controlling for these, c-*erb*B-2 was also significantly related to survival. Table II summarises the result of the Cox regression with these three variables. Controlling for nodal status, tumour size and c-*erb*B-2 status, there was no significant association between oestrogen receptor status and survival ($\chi^2 = 0.08$, 1 d.f., $P = 0.78$).

None of the possible pairwise or higher order interaction terms were statistically significant, implying that the effect of c-*erb*B-2 expression on survival is similar over the various prognostic groups defined by combinations of nodal status and tumour size.

*Relationship between grade of staining and survival*

Degree of staining was evaluated on a scale of 1 to 4 based on the percentage of cells stained. Eighteen percent were grade 1, 27% grade 2, 31% grade 3 and 23% grade 4. When the duration of survival of these four groups was compared no significant difference was observed.

| | | | | | | | | |
|---|---|---|---|---|---|---|---|---|
| | A | 62 | 55 | 45 | 38 | 36 | 23 | 4 |
| Nos at | B | 232 | 215 | 187 | 169 | 151 | 100 | 31 |
| risk | C | 41 | 28 | 21 | 17 | 16 | 8 | 1 |
| | D | 124 | 104 | 80 | 63 | 51 | 35 | 10 |

**Figure 2** Relationship of c-*erb*B-2 to survival in nodal sub-groups.

| | | | | | | | | |
|---|---|---|---|---|---|---|---|---|
| | A | 51 | 43 | 34 | 27 | 25 | 13 | 1 |
| Nos at | B | 48 | 38 | 31 | 28 | 27 | 18 | 4 |
| risk | C | 203 | 187 | 156 | 137 | 121 | 85 | 19 |
| | D | 146 | 125 | 105 | 90 | 78 | 48 | 21 |

**Figure 3** Relationship of c-*erb*B-2 to survival in oestrogen receptor sub-groups.

**Table II** Regression coefficients for the Cox proportional hazards model for cancer-related death

| *Variable* | *Coefficient* | *Standard error (SE)* | *Coefficient SE* | *P-value* |
|---|---|---|---|---|
| Nodal status* | 0.793 | 0.147 | 5.38 | <0.0001 |
| Tumour size | | | | |
| $T_1$ | −0.822 | 0.322 | −2.55 | |
| $T_2$ | −0.505 | 0.166 | −3.04 | 0.003 |
| $T_3$ | 0.0 | – | – | |
| c-*erb*B-2* | 0.327 | 0.166 | 1.97 | 0.05 |

*Coded; 0 = negative, 1 = positive.

### Discussion

The purpose of this investigation was to establish the level of expression and clinical significant of the proto-oncogene c-*erb*B-2 in patients presenting with primary breast cancer. We found that the c-*erb*B-2 receptor was expressed in 22% of patients. Although this level of expression is similar to that noted in other series it is not as high as that reported by some (Slamon *et al.*, 1987, 1989). It has been suggested that archival paraffin-embedded material may under-represent expression because antigens may be lost during storage (Slamon *et al.*, 1989). In order to evaluate this problem a separate study (results not reported) was undertaken with freshly collected tissue fixed in methacarn and stained soon after fixation in exactly the same way as the archival tissue. A similar level of expression was seen in this group to that in the archival group. Moreover, the same results were obtained with an entirely separate monoclonal antibody to c-*erb*B-2. Similarly, although multiple serial sections were not stained routinely, at least two sections from the same histological blocks were stained giving almost identical results, and the consistency of findings in those cases in which staining of multiple sections was undertaken was such that heterogeneity of expression of c-*erb*B-2 is not affecting the reported results.

The expression of c-*erb*B-2 in this study is not associated with other tumour variables, except perhaps a weak trend towards an association with larger tumours. The differences between those studies in which statistically significant association has been noted between the expression of c-*erb*B-2 and such variables as both nodal involvement (Slamon *et al.*, 1987; Zhou *et al.*, 1987) and histological grade (Berger *et al.*, 1988) may reflect differences in the characteristics of the populations of tumours considered. In this study 70% of the tumours were T2 tumours and 60% were from patients without involved lymph nodes. Other studies in which associations have been observed with both tumour size and nodal status have consisted of a much higher proportion of patients with axillary metastases or larger tumours (Slamon *et al.*, 1989). With such a relatively small percentage of tumours expressing the c-*erb*B-2 receptor, small changes in the numbers expressing the receptor result in marked changes in statistical significance, for even moderately sized groups. This underlines the importance of studying large numbers of patients when evaluating the prognostic usefulness of the c-*erb*B-2 receptor.

Overall survival for patients with tumours expressing the c-*erb*B-2 receptor was significantly worse than in those with tumours not expressing it. This poor prognostic effect was observed throughout the period of follow-up, and these findings are similar to those observed in other studies (Slamon *et al.*, 1989; Wright *et al.*, 1989*a*; Lovekin *et al.*, 1989).

Of particular clinical interest is the observation that the presence of c-*erb*B-2 in tumours may identify a sub-group of node negative patients with a poorer prognosis, and this has been observed in other studies (Wright *et al.*, 1989*a*); Richner *et al.*, 1990). The ability to distinguish patients within this ostensibly low risk group who are subject to early relapse of their disease is of potential practical value for the selection of patients for entry into adjuvant chemotherapy trials. The expression of the c-*erb*B-2 receptor in primary tumour has been shown to be associated with poor survival in patients when treated with tamoxifen at time of recurrence (Wright *et al.*, 1989*b*). The presence of c-*erb*B-2 may therefore be of use in selecting patients who may not be suitable for treatment with tamoxifen alone without the use of surgery.

Although this study indicates that the presence of c-*erb*B-2 receptor protein appears to affect prognosis in primary breast cancer, several important questions remain to be answered both in relation to its clinical utility and to its biological role in breast cancer. In pilot studies on ductal carcinoma *in situ* (DCIS) the percentage of patients with tumours expressing the c-*erb*B-2 receptor is apparently higher (42%) than in established invasive breast cancer and is associated particularly with those of the commedo pattern (van der Vijver *et al.*, 1988; Barnes *et al.*, 1988). Because this particular histological type is also associated with more aggressive tumour behaviour it has been suggested that c-*erb*B-2 may be involved in driving proliferation in the early stages of the disease (van de Vijver *et al.*, 1988). However, all *in situ* lesions may not progress to invasive lesions or alternatively expression of this gene is retained in only that proportion of tumours which grow at faster rates. Thus the presence of this receptor in small and *in situ* carcinomas may be of even more value as a prognostic indicator than in established breast carcinomas.

The mechanism by which the c-*erb*B-2 oncogene induces malignant change are not fully understood. However, the mutated form of the *neu* gene that induces malignant change in transgenic mice is not observed in human breast carcinomas (Lemoine *et al.*, 1990) and it probably requires activation by an external ligand for expression of the neoplastic phenotype in breast cancer. This ligand may be a novel growth factor which, after binding, modifies the response of cells expressing the receptor to such factors as tumour necrosis factor (Hudziack *et al.*, 1989) or part of a cell–cell signalling apparatus (Gusterson *et al.*, 1988; Quirke *et al.*, 1989).

Our results indicate that the proto-oncogene c-*erb*B-2 is associated with a poor prognosis in breast cancer. It remains to be determined what clinical significance it has in early and *in situ* breast cancers and what role it plays in the development of the disease.

### References

ALI, I.U., CAMPBELL, G., LIDEREAU, R. & CALLAHAN, R. (1988). Lack of evidence for the prognostic significance of c-*erb*B-2 amplification in human breast carcinoma. *Oncogene Res.*, **3**, 139.

BARGMANN, C.I., HUNG, M.-C. & WEINBERG, R.A. (1986). Multiple independent activations of the *neu* oncogene by a pont mutation altering the transmembrane domain of p185. *Cell*, **45**, 649.

BARNES, D.M., LAMMIE, G.A., MILLIS, R.R., GULLICK, W.J., ALLE, D.S. & ALTMAN, D.G. (1988). An immunohistochemical evaluation of c-*erb*B-2 expression in human breast cancer. *Br. J. Cancer*, **58**, 448.

BERGER, M.S., LOCHER, G.W., SAURER, S. & 4 others (1988). Correlation of c-*erb*B-2 gene amplification and protein expression in human breast carcinoma with nodal status and nuclear grading. *Cancer Res.*, **48**, 1238.

COOKE, T., GEORGE, W.D., SHIELDS, R., MAYNARD, P. & GRIFFITHS, K. (1979). Oestrogen receptors and prognosis in early breast cancer. *Lancet*, **ii**, 995.

COUSSENS, L., YANG-FENG, T.L., LIAO, Y.-C. & 7 others (1985). Tyrosine kinase receptor with extensive homology to EGF receptor shares chromosomal location with *neu* oncogene. *Science*, **230**, 1132.

GUSTERSON, B.A., MACHIN, L.G., GULLICK, W.J. & 6 others (1988). c-*erb*B-2 expression in benign and malignant breast disease. *Br. J. Cancer*, **58**, 453.

HUDZIAK, R.M., LEWIS, G.-D., WINGET, M., FENDLY, B.M., SHEPARD, H.M. & ULLRICH, A. (1989). $p185^{Her2}$ monoclonal antibody has antiproliferative effects *in vitro* and sensitised human breast tumour cells to tumour necrosis factor. *Mol. Cell Biol.*, **9**, 1165.

KRAUS, M.H., POPESCU, N.C., AMSBAUGH, S.C. & KING, C.R. (1987). Overexpression of the EGF-receptor related proto-oncogene *erb*B-2 in human mammary tumour cell lines by different molecular mechanisms. *EMBO J.*, **6**, 605.

LEMOINE, N.R., STADDON, S., DICKSON, C., BARNES, D.M. & GULLICK, W.J. (1990). Absence of activating transmembrane mutations in the c-*erb*B-2 proto-oncogene in human breast cancer. *Oncogene*, **5**, 237.

LOVEKIN, C., ELLIS, I.O., LOCKER, A. & 5 others (1989). c-*erb*B-2 oncogene expression in breast cancer: relationships and prognostic significance. *J. Pathol.*, **158**, 345A.

PHADY, L.C., SHIH, C., COWING, D., FINKELSTEIN, R. & WEINBERG, R.A. (1982). Identification of a phosphoprotein specifically induced by the transforming DNA of rat neuroblastomas. *Cell*, **28**, 865.

QUIRKE, P., PICKELS, A., TUZI, N.L., MOHAMDEE, O. & GULLICK, W.J. (1989). Pattern of expression of c-*erb*B-2 onco-protein in human foetuses. *Br. J. Cancer*, **60**, 64.

RICHNER, J., GERBER, H.A., LOCHER, G.W. & 6 others (1990). c-*erb*B-2 protein expression in node negative breast cancer. *Annals Oncol.*, **1**, 263.

SLAMON, D.J., CLARK, G.M., WONG, S.G., LEVIN, W.J., ULLRICH, A. & MCGUIRE, W.L. (1987). Human breast cancer: correlation of relapse and survival with amplification of the HER-2/*neu* oncogene. *Science*, **235**, 177.

SLAMON, D.J., GOLDOLPHIN, W., JONESHOLD, J.A. & 7 others (1989). Studies on HER-2.*neu* proto-oncogene in human breast and ovarian cancer. *Science*, **244**, 707.

VAN DE VIJVER, M., VAN DE BERSSELAAR, R., DEVILEE, P., CORNELISSE, C., PETERSE, J. & NUSSE, R. (1987). Amplification of the linked c-*erb*A oncogene. *Mol. Cell Biol.*, **7**, 2019.

VAN DE VIJVER, M.J., PETERSE, J.L., MOOI, W. & 4 others (1988). Neu-protein overexpression in breast cancer. *N. Engl. J. Med.*, **319**, 1239.

VARLEY, J.M., SWALLOW, J.E., BRAMMAR, W.J., WHITTAKER, J.L. & WALKER, R.A. (1987). Alterations to either c-*erb*B-2 (*neu*) or c-*myc* proto-oncogenes in breast carcinomas correlate with poor short term prognosis. *Oncogene*, **1**, 423.

VENTER, D.J., KUMAR, S., TUZI, N. & GULLICK, W.J. (1987). Overexpression of the c-*erb*B-2 oncoprotein in human breast carcinomas: immunohistochemical assessment correlated with gene amplification. *Lancet*, **ii**, 69.

WRIGHT, C., ANGUS, NICHOLSON, S. & 6 others (1989*a*). Expression of c-*erb*B-2 oncoprotein: a prognostic indicator on breast cancer. *Cancer Res.*, **49**, 2087.

WRIGHT, C., NICHOLSON, S., ANGUS, B. & 5 others (1989*b*). Association of c-*erb*B-2 oncoprotein expression with lack of responses to endocrine therapy in recurrent breast cancer. *J. Pathol.*, **158**, 350.

ZHOU, D., BATTIFORA, H., YOKATA, J., YAMAMOTO, T. & CLINE, M.J. (1987). Association of multiple copies of the c-*erb*B-2 oncogene with the spread of breast cancer. *Cancer Res.*, **47**, 6123.

ZHOU, D.-J., AHUJA, H. & CLINE, M.J. (1989). Proto-oncogene abnormalities in human breast cancer: c-*erb*B-2 amplification does not correlate with recurrence of disease. *Oncogene*, **47**, 6123.